# Deregulation of Biologically Significant Genes and Associated Molecular Pathways in the Oral Epithelium of Electronic Cigarette Users

**DOI:** 10.3390/ijms20030738

**Published:** 2019-02-10

**Authors:** Stella Tommasi, Andrew W. Caliri, Amanda Caceres, Debra E. Moreno, Meng Li, Yibu Chen, Kimberly D. Siegmund, Ahmad Besaratinia

**Affiliations:** 1 Department of Preventive Medicine, USC Keck School of Medicine, University of Southern California, M/C 9603, Los Angeles, CA 90033, USA; tommasi@med.usc.edu (S.T.); Andrew.Caliri@med.usc.edu (A.W.C.); Amanda.Caceres@med.usc.edu (A.C.); Debra.Moreno@med.usc.edu (D.E.M.); kims@usc.edu (K.D.S.); 2 USC Libraries Bioinformatics Service, University of Southern California, NML 203, M/C 9130, Los Angeles, CA 90089, USA; mengli2@usc.edu (M.L.); yibuchen@usc.edu (Y.C.)

**Keywords:** carcinogenesis, gene regulation, transcription, RNA-seq, vaping

## Abstract

We have investigated the regulation of genes and associated molecular pathways, genome-wide, in oral cells of electronic cigarette (e-cigs) users and cigarette smokers as compared to non-smokers. Interrogation of the oral transcriptome by RNA-sequencing (RNA-seq) analysis showed significant number of aberrantly expressed transcripts in both e-cig users (vapers) and smokers relative to non-smokers; however, smokers had ~50% more differentially expressed transcripts than vapers (1726 versus 1152). Whereas the deregulated transcripts in smokers were predominately from protein-coding genes (79% versus 53% in vapers), nearly 28% of the aberrantly expressed transcripts in vapers (versus 8% in smokers) belonged to regulatory non-coding RNAs, including long intergenic non-coding, antisense, small nucleolar and misc RNA (*P* < 0.0001). Molecular pathway and functional network analyses revealed that “cancer” was the top disease associated with the deregulated genes in both e-cig users and smokers (~62% versus 79%). Examination of the canonical pathways and networks modulated in either e-cig users or smokers identified the “Wnt/Ca^+^ pathway” in vapers and the “integrin signaling pathway” in smokers as the most affected pathways. Amongst the overlapping functional pathways impacted in both e-cig users and smokers, the “Rho family GTPases signaling pathway” was the top disrupted pathway, although the number of affected targets was three times higher in smokers than vapers. In conclusion, we observed deregulation of critically important genes and associated molecular pathways in the oral epithelium of vapers that bears both resemblances and differences with that of smokers. Our findings have significant implications for public health and tobacco regulatory science.

## 1. Introduction

Electronic cigarettes (e-cigs) are battery-powered handheld devices that simulate tobacco smoking [1]. E-cigs heat a solution (i.e., e-liquid/e-juice) containing a mixture of propylene glycol, vegetable glycerin, concentrated flavors, and optionally, variable concentrations of nicotine into inhalable vapor [2]. E-cig use is commonly referred to as “vaping”, and e-cig users are interchangeably termed “vapers” [3]. E-cigs were introduced into the US market over a decade ago as a putatively less-harmful tobacco substitute [1]. Over the past several years, the appeal and popularity of e-cigs have significantly increased as evidenced by the nearly 10-fold rise in the prevalence of vaping, especially among adult smokers [4] and adolescent never smokers [5]. Meanwhile, the number and type of e-cig products have increased exponentially, albeit little or no systematic regulation of sales has been in place [1]. In 2013–2014, 5.5% of the US adult population were the current e-cig users then, representing over 13 million people [4]. Although recent trends suggested decreasing past-month’s use of e-cigs by adolescents, the use of e-cigs in this population exceeded that of conventional cigarettes in 2015, 2016, and 2017 [6]. Furthermore, US retail sales for e-cigs have consistently increased in the past several years, and are expected to surpass those of combustible cigarettes by year 2023 [1,3].

E-cig use is a pressing public health concern in many parts of the world [1]. This is due to the uncertainties surrounding the potential health consequences of vaping and its effectiveness as a putative tobacco harm-reduction strategy [3]. Currently, there is a paucity of data on e-cig safety, and very limited scientific evidence to support the efficacy of vaping in aiding smoking cessation [1]. There is also a concern that e-cig use may lead to nicotine addiction and smoking, especially among youth [7,8]. The existing data show that e-cig vapor is not merely “water vapor” as is often claimed in alluring advertisements and marketing campaigns [2]. Chemical analyses of e-cig vapor and liquid have confirmed the presence of many toxicants and carcinogens as those found in cigarette smoke [1,2,3,9]. Although the concentrations of most carcinogenic compounds in e-cig products are much lower than those in cigarette smoke, there is no “safe” level of exposure to carcinogens [3]. Thus, lower levels of carcinogens in e-cig products do not equate to no cancer risk. It is, therefore, important to investigate whether e-cig-derived carcinogens pose a cancer risk to regular vapers and/or to those who are involuntarily exposed to e-cig contaminants in the environment. Equally important is to determine the magnitude of cancer risk associated with vaping as compared to smoking.

Human cancer is characterized by deregulation of genes involved in crucial cellular functions, such as growth control and differentiation [10,11]. Investigating the global expression of genes and associated molecular pathways and gene networks in tissues or organs of humans exposed to carcinogens can provide insights into the biological consequences of exposure to carcinogenic compounds. Consistent with tobacco smoking being a major risk factor for various types of human cancer, deregulation of cancer-related genes and disruption of associated pathways and networks have been demonstrated in target and relevant surrogate tissues of cigarette smokers [12,13,14,15]. To date, however, the impact of e-cig use on gene regulation has not been investigated in regular vapers. In the present study, we have determined the effects of vaping versus smoking on gene regulation by interrogating the oral transcriptome in exclusive e-cig users and cigarette smokers as compared to non-smokers. The choice of oral epithelial cells to evaluate the cancer-causing potential of vaping versus smoking is highly relevant because oral epithelium is a major target tissue for smoking-associated cancer [16,17]. Here, we have used a validated non-invasive brushing technique [18] to obtain oral epithelial cells from three groups of healthy adults, including exclusive e-cig users, cigarette smokers only, and non-smokers (*n* = 42, 24, and 27, respectively). We have performed whole transcriptome analysis on total RNA isolated from oral cells of the study subjects using RNA-sequencing (RNA-seq) technology. Furthermore, we have performed gene ontology analysis on the identified differentially expressed genes in e-cig users and smokers using a combination of bioinformatics resources and tools. Finally, we have validated the results, at single gene level, using reverse transcription quantitative polymerase chain reaction (RT-qPCR) analysis.

## 2. Results

### 2.1. Genome-Wide Gene-Expression Analysis

**Figure 1:**
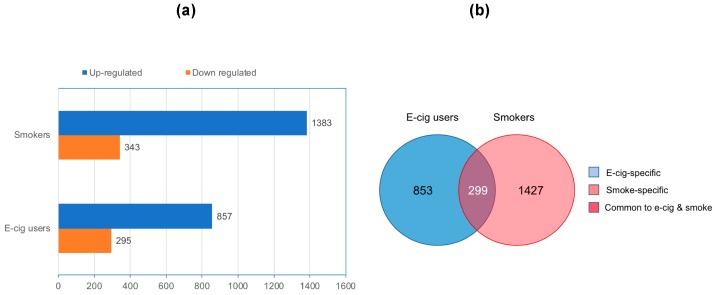
Aberrantly expressed transcripts detected by RNA-sequencing (RNA-seq) in electronic cigarette (e-cig) users and smokers as compared to controls. (**a**) Numbers of up-regulated and down-regulated transcripts in e-cig users and smokers are indicated. Fold-change: >1.5; *P* < 0.005. (**b**) Venn diagram of deregulated transcripts in e-cig users and smokers is shown.

To investigate the impact of vaping versus smoking on the whole transcriptome, we performed RNA-seq analysis on total RNA isolated from oral cells of e-cig users and cigarette smokers in comparison to controls, i.e., non-smokers non-vapers. As shown in Figure 1a, there were large numbers of differentially expressed transcripts in both e-cig users and cigarette smokers relative to controls (>1.5 fold-change and *P* < 0.005), although, smokers had nearly 50% more aberrantly expressed transcripts than e-cig users (1726 versus 1152). There were 857 up-regulated transcripts and 295 down-regulated transcripts in e-cig users, corresponding to 74.4% and 25.6% of all differentially expressed transcripts in this group. The corresponding numbers of over-expressed and under expressed transcripts in smokers were 1383 and 343, representing 80.1% and 19.9%, respectively, of all their differentially expressed transcripts. Compiled lists of aberrantly expressed transcripts and associated genomic loci (if annotated) in the e-cig users and cigarette smokers are provided in Supplementary Tables S1 and S2, respectively.

The differentially expressed transcripts in e-cig users and smokers can be classified into three categories: (I) vape-specific: transcripts exclusively deregulated in e-cig users; (II) smoke-specific: transcripts exclusively deregulated in smokers; and (III) common to vape and smoke: transcripts deregulated in both e-cig users and smokers (Figure 1b). Whereas the vape-specific transcripts comprised 74.1% of all differentially expressed transcripts in e-cig users, smoke-specific transcripts constituted 82.7% of all aberrantly expressed transcripts in cigarette smokers. The commonly deregulated transcripts in e-cig users and smokers comprised 25.9% and 17.3% of all differentially expressed transcripts in the respective groups.

Altogether, these data indicate that e-cig users have significant over-expression and under expression of genes in oral epithelium, which is a major target site for smoking-associated carcinogenesis [16,17]. The aberrantly expressed transcripts detected in e-cig users are partly overlapping with but mostly different from those found in smokers.

### 2.2. Gene Ontology and Molecular Pathway and Functional Network Analyses

**Figure 2:**
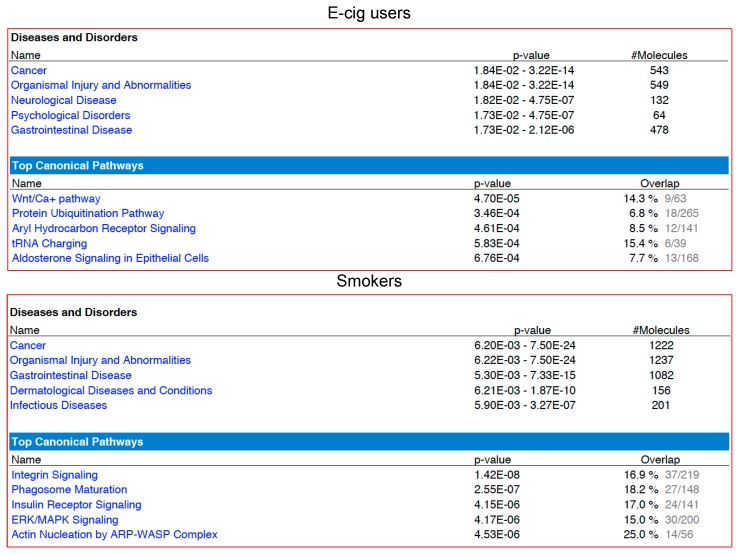
Functional pathway analysis of differentially expressed genes in e-cig users and smokers relative to controls by IPA^®^. In both groups, differentially expressed genes are predominantly associated with “cancer”. The “Wnt/Ca^+^ pathway” in e-cig users and the “integrin signaling pathway” in smokers are the most affected pathways.

**Figure 3:**
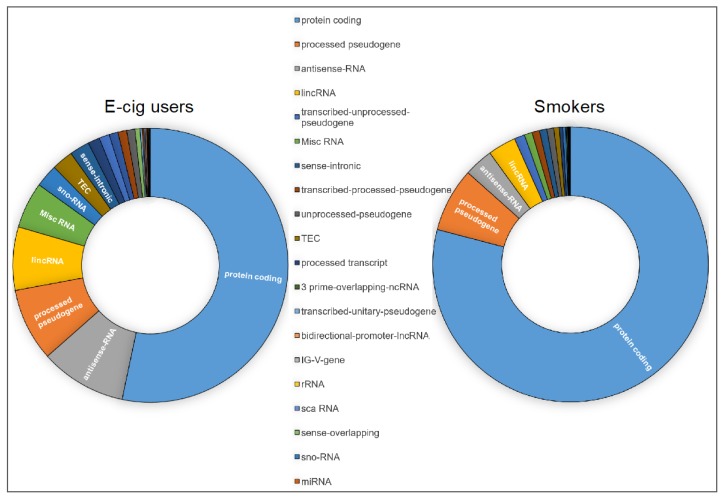
Categories of the aberrant transcripts detected in e-cig users and smokers relative to controls by IPA^®^. High proportions of the aberrantly transcribed DNA sequences in e-cig users and smokers, respectively, belong to regulatory non-coding RNAs and protein-coding genes.

We next used a combination of the Ingenuity Pathway Analysis^®^ (IPA^®^ v. 9.0) and the gene ontology (GO) functional annotation clustering analysis (Database for Annotation, Visualization and Integrated Discovery (DAVID) v. 6.8) to obtain a detailed gene ontology information on the gene lists generated by RNA-seq in e-cig users and smokers as compared to controls. Of the 1152 aberrantly expressed transcripts in e-cig users, 876 (76%) mapped to known IDs in the IPA database, whereas 1539 out of 1726 deregulated transcripts in smokers (89%) had an assigned ID. As shown in Figure 2, cancer was the top listed disease associated with the deregulated targets in both e-cig users (543 out of 876 identified transcripts: ~62%) and smokers (1222 out of 1539 identified transcripts: ~79%). Of significance, only 53% of the aberrantly transcribed DNA sequences in e-cig users versus 79% in smokers were protein-coding (*P* < 0.0001) (Figure 3). On the other hand, nearly 28% of the aberrant transcripts detected in e-cig users belonged to diverse classes of regulatory non-coding RNAs, including long intergenic non-coding (linc), antisense, small nucleolar (sno), and misc RNA (Figure 3). In smokers, however, a much smaller percentage of differentially expressed transcripts (~8%) consisted of regulatory non-coding RNAs (*P* < 0.0001) (Figure 3).

**Figure 4:**
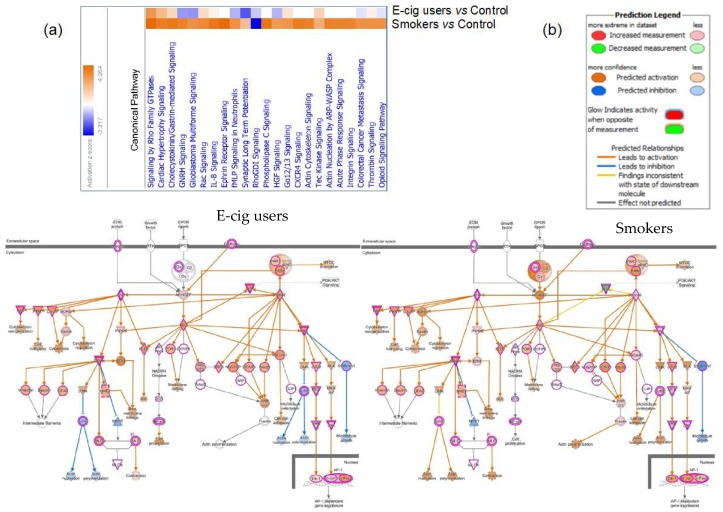
Common canonical pathways disrupted in e-cig users and smokers relative to controls by IPA^®^. (**a**) The canonical pathway heatmap visualizes the affected canonical pathways simultaneously in e-cig users and smokers, allowing a direct comparison between the two groups. (**b**) Predicting how up-regulated and down-regulated genes in the datasets (red and green nodes, respectively, on the pathway) can affect the activity of other molecules on the pathway. Orange nodes, prediction of activation; blue nodes, prediction of inhibition.

To further compare and contrast the molecular effects associated with vaping and smoking, we examined the canonical pathways and networks that were modulated in either e-cig users or smokers. We found that the most affected pathway in e-cig users was the “Wnt/Ca^+^ pathway” (*P* = 4.7 × 10^−5^), while in smokers the “integrin signaling pathway” was primarily impacted (*P* = 1.42 × 10^−8^) (Figure 2). To explore possible commonalities between the biological effects of vaping and smoking, we also examined the overlapping functional pathways that were compromised in both e-cig users and smokers as compared to non-users. The heatmap generated by Comparison Analysis in IPA summarizes all the significant signaling cascades detected across the two datasets (Figure 4a). As shown in Figure 4a, the “Rho family GTPases signaling pathway” is the top deregulated pathway in both vapers and smokers, although the number of affected targets is three times higher in smokers than vapers (27 versus 9). The Molecule Activity Predictor (MAP) tool was used to predict how the up-regulated and down-regulated genes in the datasets could affect the activity of other molecules in the Rho family GTPases signaling cascade (Figure 4b). The Comparison Analysis in IPA was also used to identify upstream regulators, including transcription factors and chemicals, whose activities appeared to be affected in both e-cig users and smokers, at times with opposing effects (Figure S1). Amongst the identified upstream regulators is the tumor suppressor p53 gene (*TP53*), which is the most frequently mutated gene in head and neck squamous cell carcinoma-associated with smoking (41%) [19] (Figure S2).

**Figure 5:**
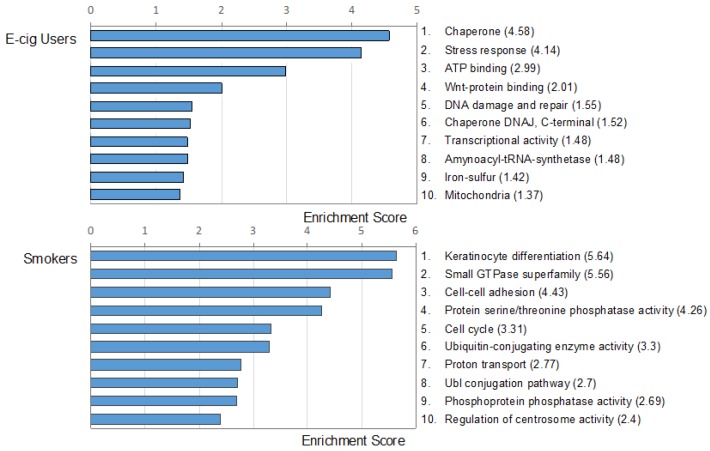
Gene ontology analysis of the differentially expressed genes in e-cig users and smokers relative to controls by Database for Annotation, Visualization and Integrated Discovery (DAVID). The top ten functional clusters in e-cig users and smokers are shown. Enrichment scores are indicated in parentheses.

DAVID analysis of the datasets generated by RNA-seq in e-cig users and smokers confirmed the gene ontology results obtained by IPA. Using Functional Annotation Clustering Enrichment Score in DAVID, we identified top clusters in e-cig users and smokers as compared to controls (Figure 5). The most represented functional categories in e-cig users included molecules involved in chaperones, stress response, and ATP-binding and Wnt-binding proteins. Of note, many of the DAVID IDs identified in e-cig users are known to be specifically involved in tumorigenesis, particularly, in smoking-related cancer, such as lung cancer, squamous cell carcinoma of the head and neck, esophageal cancer, bladder cancer, ovarian cancer, and leukemia (see, Table S3). In smokers, the top functional clusters included genes involved in keratinocyte differentiation, small GTPase superfamily, cell–cell adhesion, and protein serine/threonine phosphatase activity (Figure 5).

Altogether, our gene ontology analyses show that vapers, similarly to smokers, have deregulation of key genes, the majority of which being cancer-related and potentially involved in the initiation and/or promotion of tumorigenesis. Whilst the extent of gene deregulation in vapers differs from that of smokers, the disrupted functional pathways and networks in the vapers are partly similar to, but mostly different from those found in smokers.

### 2.3. Validation of Gene-Expression Data by Real-Time Reverse Transcription Quantitative PCR (RT-qPCR)

**Figure 6:**
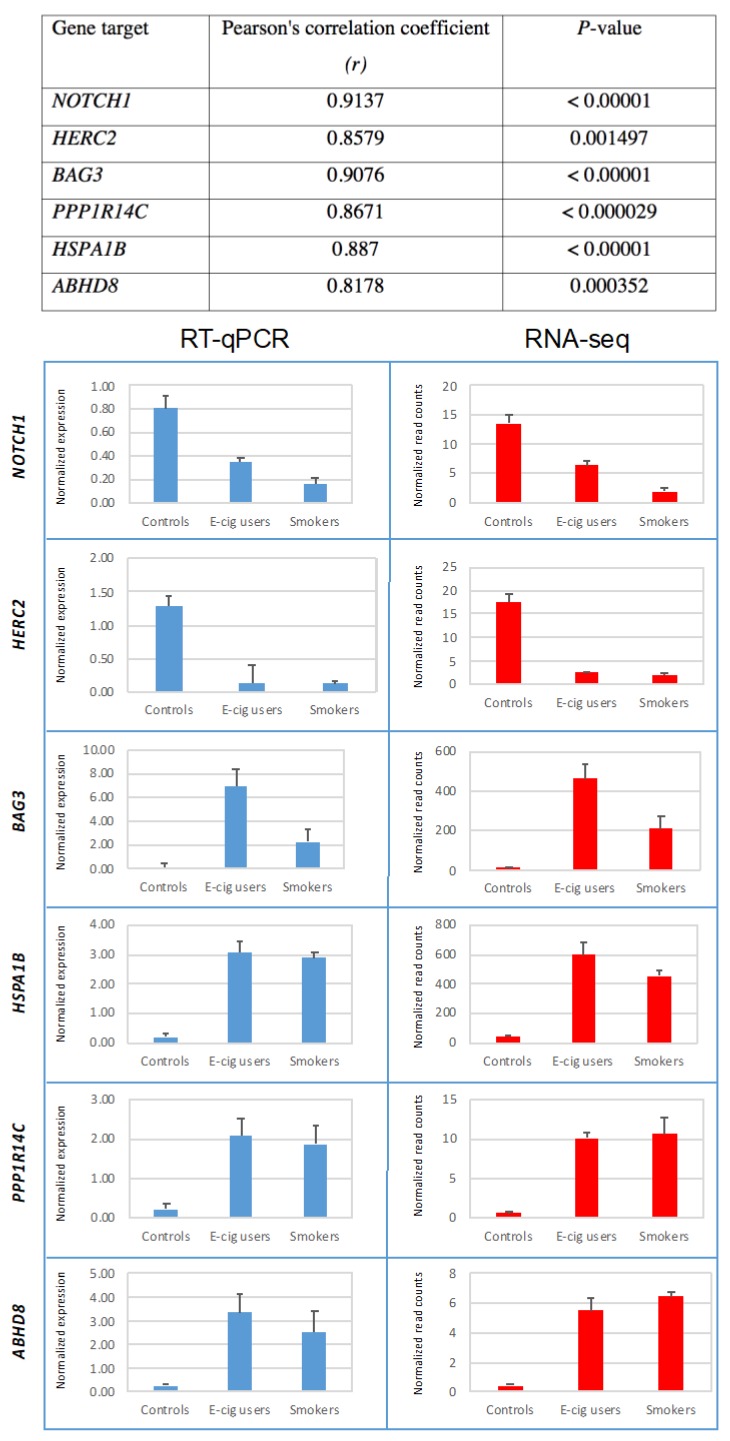
Validation of RNA-seq gene-expression data by RT-qPCR. Transcription levels of several up- and down-regulated targets identified by RNA-seq in e-cig users and smokers were validated by RT-qPCR. Results are expressed as the median normalized expression levels + Standard Error (SE).

To independently validate the gene-expression data, we randomly selected several up- and down-regulated targets identified by RNA-seq in the e-cig user and smoker groups, and examined their transcription levels by RT-qPCR. Figure 6 shows the median normalized expression levels of several aberrantly expressed transcripts initially detected by RNA-seq in the oral cells of e-cig users and smokers, as compared to controls. In agreement with the RNA-sequencing results, we detected down-regulation of two tumor suppressor genes, including *NOTCH1* and *HERC2* [20,21], in both e-cig users and smokers (Figure 6). Furthermore, we verified up-regulation of the BCL2 associated athanogene 3 (*BAG3*) gene and its binding partner, the heat shock protein 70 (*HSPA1B*), the protein phosphatase 1 regulatory inhibitor subunit 14C (*PPP1R14C*), and abhydrolase domain containing 8 (*ABHD8*) in both e-cig users and smokers (Figure 6). In all cases, we observed statistically significant correlations between expression levels of genes detected by RNA-seq and their corresponding expression levels quantified by RT-qPCR (see Figure 6).

Altogether, we have confirmed deregulation of a number of gene targets initially identified by RNA-seq analysis in e-cig users and smokers. More specifically, we observed a high degree of correlation between expression levels of aberrant transcripts detected by RNA-seq analysis and their corresponding expression levels measured by RT-qPCR, which attests to the validity and reproducibility of our whole transcriptome analysis.

### 2.4. Verification of Smoking/Vaping Status

**Figure 7:**
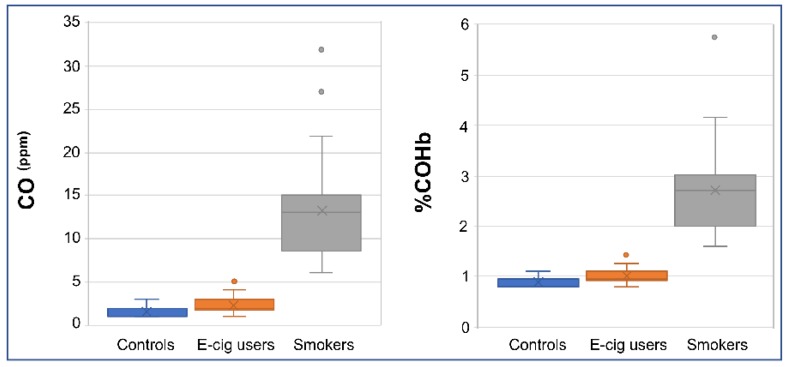
Validation of smoking/vaping status. Exhaled breath was measured in e-cig users, smokers, and controls (*n* = 18, 25, and 30, respectively) by a Bedfont Micro^+TM^ Smokerlyzer^®^ Breath CO monitor according to the instructions of the manufacturer (Bedfont Scientific Ltd., Kent, UK). Whisker box plots display distributions of carbon monoxide (CO) levels (parts per million, ppm) and percentage of carboxyhemoglobin (%COHb) in the respective groups.

We measured the concentration of plasma cotinine, a prominent metabolite of nicotine [22], in e-cig users and cigarette smokers in comparison to controls (i.e., non-smokers non-vapers) by an enzyme-linked immunosorbent assay (ELISA). As shown in Table 1, smokers and vapers had comparable levels of plasma nicotine, which were significantly higher than those in non-smokers (*P* < 0.001). Measuring carbon monoxide (CO) levels in smokers’ breath provides an objective biomarker of recent exposure to tobacco smoke [23,24]. Therefore, we also measured CO levels in exhaled breath from subgroups of e-cig users, smokers, and controls by a breath CO monitor. Whereas smokers had significantly higher levels of breath CO as compared to controls (*P* < 0.00001), e-cig users showed almost similar levels of breath CO to those of controls (Figure 7). The estimated percentage of carboxyhemoglobin (%COHb), which is indicative of the proportion of red blood cells carrying CO instead of oxygen [25], was significantly higher in smokers than controls (*P* < 0.00001). Conversely, e-cig users and controls had approximately similar %COHb (Figure 7). Our measurements of plasma cotinine and breath CO, and determination of %COHb were fully consistent with smoking/vaping status of the study participants as reported in their questionnaires and personal interviews. This reassures the validity of our screening tools to reliably determine the smoking/vaping status of the study participants.

## 3. Discussion

We have investigated the regulation of genes and associated molecular pathways, genome-wide, in oral cells of exclusive e-cig users and cigarette smokers as compared to non-smokers. The use of oral epithelial cells to evaluate the biological consequences of vaping versus smoking relevant to cancer is significant due to the following reasons: (i) over 90% of all human cancers are of epithelial origin [26]; (ii) oral epithelium is the first site of exposure to carcinogens present in both e-cig vapor and cigarette smoke [27,28]; (iii) oral epithelial cells are a major target for tumor development and other anomalies associated with tobacco use [16,17]; (iv) oral epithelium possesses xenobiotic enzymes capable of converting proximate carcinogens to reactive metabolites [29,30]; (v) oral epithelial cells and lung epithelial cells show striking similarities in response to tobacco carcinogens, as evidenced by the comparable patterns of genotoxic [31,32,33,34] and transcriptomic effects [14,15,30,35,36] in oral cells and lung cells, respectively, from smokers; and (vi) oral epithelial cells are readily available for sampling by non-invasive techniques [36,37].

Interrogation of the whole transcriptome in oral cells of vapers and smokers as compared to non-smokers by RNA-seq analysis identified significant number of aberrantly expressed transcripts in both e-cig users and cigarette smokers, although, smokers had approximately 50% more differentially expressed transcripts than vapers (1726 versus 1152; see Figure 1). We obtained a detailed gene ontology information on the aberrantly expressed genes detected in e-cig users and smokers relative to controls using a combination of bioinformatics resources and tools. As shown in Figure 2, “cancer” was the top disease associated with the deregulated genes in both e-cig users and smokers (~62 versus 79%). Whereas the deregulated transcripts in smokers are predominately from protein-coding genes (79 versus 53% in vapers; *P* < 0.0001), more than a quarter of the aberrantly expressed transcripts in e-cig users belong to several classes of regulatory non-coding RNAs (Figure 3). An increasing number of reports has documented the crucial role of non-coding RNAs in a variety of biological and pathological processes. Among other things, functional non-coding RNAs are known to regulate the expression of protein-coding genes, and are involved in the maintenance of genome integrity [38,39]. Of relevance to this study, several long non-coding and small nucleolar RNAs have been found to be deregulated in head and neck squamous cell carcinoma [40,41], a cancer type strongly associated with tobacco smoking [42]. It has been suggested that these regulatory non-coding RNAs may drive malignant transformation by influencing cancer cell viability and motility [40,41].

Examination of the canonical pathways and networks that were modulated in either e-cig users or smokers showed that two prominent signaling pathways, including the “Wnt/Ca^+^ pathway” in vapers and the “integrin pathway” in smokers, were the most affected pathways (see Figure 2). The Wnt/Ca^2+^ signaling pathway is less characterized than its canonical counterpart, the Wnt/β-catenin pathway [43]. Initially, the Wnt/Ca^+^ pathway was thought to play a unique role in development; however, there is now substantial evidence that this signaling cascade is involved in many other molecular processes [43]. For instance, it has been shown that the Wnt/Ca^+^ signaling pathway, which is activated by the tumor suppressor WNT5A in the presence of a “frizzled” class receptor, is down-regulated in several types of cancer [43]. In the present study, the WNT5A gene and the frizzled receptor FDZ7 gene were down-regulated in e-cig users, most likely causing inhibition of downstream effectors of the cascade (Table S1). The top deregulated signaling pathway in smokers, the integrin signaling pathway, is known to modulate cell proliferation, survival, and migration. When deregulated, this pathway can promote tumor invasion and metastasis [44].

Amongst the overlapping functional pathways that were impacted in both e-cig users and smokers, the “Rho family GTPases signaling pathway” was the top disrupted pathway in both vapers and smokers, although the number of affected targets was three times greater in smokers than in vapers. The GTPase family of small GTP-binding proteins comprises a group of signaling molecules that are activated by growth factors, hormones, integrins, cytokines, and adhesion molecules [45]. They regulate a wide range of biological processes, including reorganization of the actin cytoskeleton, transcriptional regulation, vesicle trafficking, morphogenesis, neutrophil activation, phagocytosis, mitogenesis, apoptosis, and tumorigenesis. One major function attributed to Rho GTPases is the organization of the actin cytoskeleton, with the formation of actin stress fibers and focal adhesion complexes. In addition, Rho GTPases may play a role in the DNA damage response following genotoxin treatment [45]. Recently, it has been proposed that these GTPases regulate structures of the nuclear cytoskeleton, assuring the temporal and spatial distribution of DNA repair factors at the site of damage [45].

We independently validated the RNA-seq data by performing RT-qPCR analysis on randomly selected deregulated genes identified by whole transcriptome analysis. Consistent with RNA-sequencing results, we verified down-regulation of two tumor suppressor genes, including *NOTCH1* and *HERC2* [20,21], in both e-cig users and smokers (Figure 6). The NOTCH signaling pathway regulates the fates of segregating daughter cells during asymmetric cell division, and plays a crucial role in the maintenance of the adult oral squamous epithelium [20]. Reduced expression of *NOTCH1* has been detected by immunohistochemistry not only in oral squamous cell carcinoma but also in oral epithelial dysplasia, suggesting that deregulation of *NOTCH1* is an early event in the disease [20]. Of relevance, loss-of-function mutations of the *NOTCH1* gene have been detected in about 10% of head and neck squamous cell carcinoma (HNSCC), making it one of the most mutated genes in squamous cell carcinoma, which is highly associated with smoking [19,46]. Moreover, we confirmed down-regulation of the HECT and RLD domain containing E3 ubiquitin ligase (*HERC2*) in e-cig users and smokers as compared to controls. The best characterized functions of *HERC2* include its involvement in DNA repair, DNA replication, and checkpoint control [21]. Apart from its role in maintaining genome integrity, *HERC2* can also function as a cell growth regulator. *HERC2* is known to modulate p53 activity by regulating its oligomerization. Depletion of HERC2 has been shown to up-regulate cell growth and increase focus formation. Frameshift mutations of *HERC2* have been reported in gastric and colorectal cancer with microsatellite instability [21].

Consistent with the RNA-seq data, we also detected up-regulation of the BCL2 associated athanogene 3 (*BAG3*) gene in both e-cig user and smoker (Figure 6). *BAG3* belongs to a family of co-chaperones characterized by a C-terminal BAG domain that binds the HSP70/HSPA ATPase domain, and thus regulates the fate of HSP70 substrates. *BAG3* is implicated in the regulation of a variety of cellular processes, including apoptosis, development, cytoskeleton arrangement, and selective macro-autophagy. *BAG3* is thought to play a key role in the development of a wide variety of diseases, including cancer [47]. Its binding partner, the heat shock protein 70 (HSPA1B), was also over-expressed in the oral cells of e-cig users and smokers in this study (Figure 6). Protein phosphorylation is a major regulatory mechanism of signal transduction cascades in eukaryotes, catalyzed by the reciprocal activity of protein kinases and phosphatases. We also analyzed the transcription status of the protein phosphatase 1 regulatory inhibitor subunit 14C (*PPP1R14C*), a major inhibitor of the Ser/Thr protein phosphatase-1 (*PP-1*) and confirmed that this gene was up-regulated in both e-cig users and smokers (Figure 6). *PPP1R14C* plays a crucial role in multiple signal transduction pathways controlling cell cycle, protein synthesis, neuronal activity, metabolism, and muscle contraction. Malfunction of these inhibitor proteins has been linked to a variety of diseases, including cancer and cardiovascular disease [48].

Lastly, we acknowledge that our study population consisted of healthy adults of both genders at different ages and of diverse races and ethnicities as well as other characteristics (see Table 1). Given the relatively small size of our study population, matching all those characteristics in the three groups of vapers, smokers, and controls would not have been feasible. For example, while smokers were older than vapers, controls and vapers had a relatively similar age distribution. This is not unexpected considering that e-cigs are a comparatively new tobacco product whereas combustible cigarettes are not. Our catchment area for this and other ongoing studies on e-cigs and smoking is the Greater Los Angeles Area, and our latest recruitment records among >2350 subjects show consistently higher median age for smokers than vapers (ongoing work). We would like to, however, stress that the differential gene-expression profile of vapers in the present study was established based on comparison with controls (i.e., non-smokers non-vapers) whose median age was not statistically significantly different from that of vapers. Currently, work in our laboratory is underway to expand our investigations to large groups of e-cig users, smokers, and controls, whose relevant characteristics are fully matched.

In summary, our whole transcriptome analysis of oral cells from exclusive e-cig users and smokers shows that vapers, similarly to smokers, have deregulation of key genes, the majority of which converging on cancer-related pathways and functions. The extent of gene deregulation and the affected pathways in e-cig users are partly overlapping with, but mostly distinct from those of smokers. Follow-up functional studies on the identified deregulated genes and associated pathways are currently underway in our laboratory. To our knowledge, this is the first report to demonstrate that e-cig users have significant deregulation of critically important genes and associated molecular pathways in the oral epithelium, which is a major target tissue for smoking-associated cancer [16,17]. Our findings warrant further investigations into the long-term effects of vaping not only in regular e-cig users but also in non-users who are involuntarily exposed to secondhand e-cig vapor, e.g., children and fetuses of vaping pregnant mothers. Evidence from research studies, such as the present one, can lay the foundation for the development of scientifically based regulations on e-cig manufacturing, marketing, and distribution.

## 4. Materials and Methods

### 4.1. Subject Recruitment and Enrollment

This study was conducted in accordance with the Declaration of Helsinki of 1975 (https://www.wma.net/what-we-do/medical-ethics/declaration-of-helsinki/), revised in 2013. The study was approved by Health Sciences Institutional Review Board of the University of Southern California (HSIRB-USC), under the protocol number HS-16-00175 (Approval date: 4 April 2016). The study was advertised in online forums, including Craigslist, Reddit, and myUSC (http://my.usc.edu), and through social media (Twitter, Instagram, and Facebook). Also, flyers and leaflets were used to advertise the study in local colleges, universities, and vape shops. Furthermore, an online survey was developed, validated, and subsequently employed to solicit and query potential participants (http://geteo.usc.edu). Individuals who appeared to have met the study criteria were contacted by phone to complete a screening questionnaire. Based on the information obtained during the phone screen, those who were deemed potentially eligible, were scheduled for an in-person visit to our laboratory. During the visit, an expanded version of the phone screen was administered to reconfirm eligibility, and informed consent was obtained, afterwards (see below).

### 4.2. Inclusion and Exclusion Criteria for the Study

Eligible candidates for the study included healthy male or female adults of diverse races and ethnicities who could read and write in English and understand and give informed consent. Health indicators for exclusion were oral infection or inflammation, gum disease, dental decay, immune system disorders, diabetes, respiratory diseases (e.g., asthma), kidney diseases, body mass index <18 kg/m^2^ or >40 kg/m^2^, or any medical disorder/medication that could affect the subject’s safety or study results. Any unstable or significant medical condition in the past 12 months, including but not limited to symptomatic heart conditions, chronic obstructive pulmonary disease (COPD), stroke, severe angina, and hypertension, was ground for exclusion. Being pregnant or having a baby in the past 12 months was also exclusionary. Other exclusion criteria included uncontrolled mental illness or substance abuse or inpatient treatment for those conditions in the past 12 months, use of recreational or illicit drugs (e.g., marijuana or cocaine) in the past 6 months, and use of any medication known to induce/inhibit CYP450 2A6 enzyme.

### 4.3. Personal Interview

Upon reconfirmation of the eligibility and obtaining informed consent, all participants underwent a personal interview to provide detailed information about demographics, socio-economic status, use of e-cigs, cigarettes, or other tobacco products; dietary habits, lifestyle, specifically, use of recreational/illicit drugs, alcohol, and prescription or over-the-counter medicine; occupational and residential history (specifically, secondhand smoke exposure); and family history of disease. Per request of one of the reviewers, we confirm that, based on the questionnaire and in-person interview data, there was no significant difference in secondhand smoke exposure among e-cig users, smokers, and controls.

### 4.4. Study Population

The study population consisted of healthy adults—both males and females of different races and ethnicities, between the ages of 22 and 55 during the time of data collection—who resided in the Greater Los Angeles Area. The lower age-limit was consistent with a new California law that bans the sale of e-cigs and other tobacco products to anyone under the age of 21. The higher age-limit was to ensure inclusion of a good range of participants, while avoiding the confounding effects of advanced aging. The study population comprised of three groups: (I) exclusive e-cig users (*n* = 42), (II) cigarette smokers only (*n* = 24), and (III) control non-smokers non-vapers (*n* = 27). We note that differences in group size were taken into account during statistical analysis. Criteria for classification of subjects, as e-cig users, cigarette smokers, or controls, were as follows: e-cig users were those who reported current use of e-cigs for at least 3 times a week for a minimum of 6 months, and no use of combustible cigarettes or any other tobacco products in the past 6 months. Smokers were those who reported current smoking of tobacco cigarettes at least 3 times per week for a minimum of 1 year, and no use of any other tobacco products, including e-cigs, in the past 6 months. Controls were those who reported no use of any tobacco product (e-cigs or combustible) more than 5 times in their life (lifetime consumption: fewer than 100 cigarettes or less than 5 vaping sessions), with no use in the past 6 months. Detailed characteristics of the study population are listed in Table 1.

### 4.5. Sampling and Processing of Oral Epithelial Cells

All subjects were required to refrain from eating, smoking, or vaping, at least, 1 h prior to visiting our laboratory. Before sampling, subjects were asked to vigorously rinse their mouths with water to remove saliva, residual food particles, and mucosal debris. An Ultra Soft Oral-B brush (SENSI.SOFT™ Cincinnati, OH, USA) was placed in subject’s mouth, and sufficient pressure was applied to contact the surface of the inside of his/her cheeks. Rotatory motion along the face and edge of the brush was used to gently scrape the entire surface area of the inside of the cheek, while avoiding mouth bleeding. The proximal, central, and distal regions of the inside of each cheek were brushed 15 times each. Once brushing of a region was completed, the brush was swirled in a tube pre-filled with 35 mL ice-cold sterile phosphate buffer saline (PBS) to dislodge the cells from the bristles. Cycles of brushing and washing the cells off of the bristles were repeated until all regions from both cheeks were sampled. The two tubes containing the harvested cells from opposite cheeks were centrifuged at 800× *g* for 5 min at 4 °C. Pelleted cells from each tube were re-suspended in PBS, pooled into a single tube, and re-centrifuged as above. The collected cell pellet was snap frozen and kept at −80 °C until further analysis. In our preliminary studies, we have confirmed that this protocol provides ample number of cells, the vast majority of which being intermediate and suparbasal oral epithelial cells. To rule out significant contamination by other cell types, we have also performed differential cell count on the collected oral cells and verified the overwhelming presence of oral epithelial cells in all samples. To avoid any potential bias, sample collection and processing of the collected specimens from different groups were done in variable orders, not in batches, and in a “blind” fashion.

### 4.6. Sampling of Peripheral Blood

To verify the vaping/smoking status of the study participants, peripheral blood (30 mL) was collected by venipuncture, and plasma cotinine was measured, as described below. Plasma fraction was collected by centrifugation, and erythrocytes and granulocytes, lymphocytes, and peripheral mononuclear cells were further separated using the Leucosep™ tubes according to the manufacturer’s instructions (Greiner Bio-One Inc., Monroe, NC, USA). The collected plasma was snap frozen and preserved at −80 °C until further analysis.

### 4.7. RNA-Seq Analysis

Total RNA was isolated from snap frozen oral epithelial cells using the RNeasy Mini Kit (Qiagen, Valencia, CA, USA). The isolated RNA samples were checked for quality control using the RNA 6000 Nano Chip kit in an Agilent 2100 Bioanalyzer (Agilent Technologies, Santa Clara, CA, USA). Libraries for RNA-seq were prepared from total RNA (300 ng per sample) using the Kapa Hyper Prep Kits with RiboErase (Kapa Biosystems, Wilmington, DE, USA). The workflow consisted of rRNA depletion, cDNA generation, and end repair to generate blunt ends, A-tailing, adaptor ligation, and PCR amplification. Different adaptors were used for multiplexing samples in one lane. Sequencing was performed on Illumina Nextseq500 for a single-end read for 75 cycles. Data quality check was done on Illumina SAV. Demultiplexing was performed with Illumina Bcl2fastq2 v2.17 program. To rule out any potential bias, library construction and data acquisition for samples from different groups (vapers, smokers, and controls) were done in the same run, not in different batches, and in a “blind” fashion. Detailed descriptions of data processing and analysis are provided in Supplementary Data. The RNA-seq data will be deposited in the Gene Expression Omnibus database at NCBI (htttp://www.ncbi.nlm.nih.gov/geo/), and accession number will be provided as soon as it becomes available.

### 4.8. Gene Ontology and Canonical Pathways Analyses

Gene ontology (GO) analysis was performed using a combination of the Database for Annotation, Visualization and Integrated Discovery (DAVID) Bioinformatics Tool v.6.8 [49] and the Ingenuity^®^ Pathway Analysis (IPA^®^) v.9 tool (QIAGEN Bioinformatics, Redwood City, CA, USA; www.ingenuity.com). The Functional Clustering Analysis tool in DAVID was used to group together redundant GO categories and other types of functional terms (e.g., pathway, disease, etc.), while functional identification of gene networks, canonical pathways, and upstream regulators was done by IPA^®^.

### 4.9. Reverse Transcription Quantitative PCR (RT-qPCR)

To validate the RNA-seq data, we used our published RT-qPCR protocol with a few modifications [50] to determine the expression level of individual up-regulated or down-regulated genes identified by sequencing analysis. Briefly, total RNA (0.1–0.2 micrograms or µg) isolated from oral epithelial cell samples was reverse transcribed into cDNA using the iScript™ Reverse Transcription Supermix for RT-qPCR (iScript RT Supermix) (Bio-Rad laboratories, Inc., Hercules, CA, USA). The synthesized cDNA (20 ng) was pre-amplified for 12 cycles using a pool of custom-made primers and the SsoAdvanced™ PreAmp Supermix, according to manufacturer’s instructions (Bio-Rad laboratories, Inc.). The completed pre-amplification reaction was then diluted 1:10 with TE buffer (10 mmol/L Tris-HCl, 1 mmol/L EDTA, pH 8.0) and 2 microlites or µL of pre-amplified diluted cDNA was used per reaction in the presence of gene-specific primers and SsoAdvanced™ Universal SYBR^®^ Green Supermix (Bio-Rad laboratories, Inc.). The human TATA-binding protein (*TBP*) gene was used as a reference. All PCR reactions were carried out using the CFX96 Touch™ Real-Time PCR detection system (Bio-Rad Laboratories, Inc.). The cycling conditions included a pre-incubation at 95 °C for 2 min, followed by 40 cycles at 95 °C for 5 s and 58 °C for 30 s. All reactions (5 samples per group) were performed in triplicate for a total of 15 reactions per biological set. Fold-changes in transcript levels were calculated in the biological sets (e.g., e-cig users and smokers versus controls) using the Bio-Rad CFX Maestro™ software (Bio-Rad laboratories, Inc.). The primer sets used for RT-qPCR are available upon request.

### 4.10. Cotinine Measurement

Plasma cotinine was measured by a solid phase competitive enzyme-linked immunosorbent assay (ELISA), as described in Supplementary Data.

### 4.11. Measurement of Carbon Monoxide in Breath and Determination of %Carboxyhemoglobin

Carbon monoxide (CO) levels in exhaled breath and percentage of carboxyhemoglobin (%COHb) were determined using a Bedfont Micro^+TM^ Smokerlyzer^®^ Breath CO monitor, according to the instructions of the manufacturer (Bedfont Scientific Ltd., Kent, UK).

## Figures and Tables

**Table 1:** Characteristics of the study population.

		E-cig Users (*n* = 42)	Smokers (*n* = 24)	Controls (*n* = 27)
Age *		28 ± 1.3	42 ± 2.8	24 ± 1.7
Gender	Male	34 (81.0%)	19 (79.2%)	16 (59.3%)
	Female	8 (19.0%)	5 (20.8%)	11 (40.7%)
Race	White	16 (38.1%)	5 (20.8%)	5 (18.5%)
	Hispanic	12 (28.6%)	1 (4.2%)	5 (18.5%)
	African American	5 (11.9%)	9 (37.5%)	2 (7.4%)
	Asian	7 (16.7%)	4 (16.7%)	12 (44.4%)
	Other	2 (4.8%)	5 (20.8%)	3 (11.1%)
Education	Less than high school	0 (0%)	3 (12.5%)	0 (0%)
	High school diploma or GED	11 (26.2%)	2 (8.3%)	0 (0%)
	Some college completed or currently enrolled in college	19 (45.2%)	8 (33.3%)	1 (3.7%)
	College degree or higher	12 (28.6%)	11 (45.8%)	26 (96.3%)
Marital status	Married	6 (14.3%)	3 (12.5%)	0 (0%)
	Currently living with someone	2 (4.8%)	0 (0%)	0 (0%)
	Widowed	1 (2.4%)	0 (0%)	0 (0%)
	Separated	1 (2.4%)	1 (4.2%)	0 (0%)
	Divorced	5 (11.9%)	2 (8.3%)	3 (11.1%)
	Single and never married	27 (64.3%)	18 (75.0%)	24 (88.9%)
Employment status	Full time	26 (61.9%)	12 (50%)	19 (70.4%)
	Part time	9 (21.4%)	4 (16.7%)	6 (22.2%)
	Retired or disability	1 (2.4%)	3 (12.5%)	0 (0%)
	Unemployed	6 (14.3%)	5 (20.8%)	2 (7.4%)
Pre-tax-annual income	<$15,000	4 (9.5%)	9 (37.5%)	9 (33.3%)
	≥$15,000 to <$30,000	12 (28.6%)	5 (20.8%)	5 (18.5%)
	≥$30,000 to <$45,000	9 (21.4%)	4 (16.7%)	4 (14.8%)
	≥$45,000 to <$60,000	6 (14.3%)	1 (4.2%)	2 (7.4%)
	≥$60,000 to <$75,000	3 (7.1%)	0 (0%)	2 (7.4%)
	≥$75,000 to <$90,000	3 (7.1%)	1 (4.2%)	1 (3.7%)
	≥$90,000 to <$105,000	1 (2.4%)	1 (4.2%)	1 (3.7%)
	≥$105,000 to <$120,000	0 (0%)	1 (4.2%)	0 (0%)
	≥$120,000	4 (9.5%)	2 (8.3%)	3 (11.1%)
BMI *^,†^		27.9 ± 1.1	27.2 ± 0.9	25.0 ± 1.3
Pack Year *^,‡^		NA	12.3 ± 2.5	NA
Cumulative e-liquid (mL) *^,¶^		5369.5 ± 3038.5	NA	NA
Cumulative e-nicotine (mg) *^,¶¶^		20,859.7 ± 10,346.6	NA	NA
Plasma cotinine (ng/mL) *		115.0 ± 8.5	122.0 ± 10.8	2.5 ± 0.1

* Results are expressed as Median ± SE. ^†^ BMI, body mass index (Weight (kg) ÷ Height^2^ (m)). ^‡^ In smokers, pack year was calculated by multiplying the number of packs of cigarette a person smoked per day by the number of years he/she smoked. ^¶^ In e-cig users, cumulative e-liquid was calculated as the total volume of e-liquid (in milliliter) vaped by a person during his/her lifetime. ^¶¶^ In e-cig users, cumulative e-nicotine was calculated as the total amount of nicotine (in milligrams) present in e-liquid vaped by a person during his/her lifetime. GED, General Education Development or General Education Diploma; the GED or High School Equivalency Certificate shows that one has a level of knowledge equivalent to a high school graduate. NA, not applicable.

## Supplementary Materials

Supplementary materials can be found at http://www.mdpi.com/1422-0067/20/3/738/s1. Table S1: Compiled list of aberrantly expressed transcripts and associated genomic loci (if annotated) in the e-cig users versus controls. Table S2: Compiled list of aberrantly expressed transcripts and associated genomic loci (if annotated) in cigarette smokers versus controls. Table S3: Relevant diseases identified by DAVID in e-cig users. Figure S1: Comparison of the upstream regulators affected in both e-cig users and smokers relative to controls by IPA^®^. Figure S2: Visualization of the network of deregulated genes converging on *TP53* gene in e-cig users and smokers relative to controls by IPA^®^.

## Dedication

This work is dedicated to the memory of our beloved colleague and friend, Steven E. Bates, who was an inspiration to all of us.
