# Supplementary material for: Deregulation of Biologically Significant Genes and Associated Molecular Pathways in the Oral Epithelium of Electronic Cigarette Users
Source: Int J Mol Sci. 2019 Feb 10;20(3):738. doi: 10.3390/ijms20030738 (PMC6386888; doi:10.3390/ijms20030738)
Supplement: Supplementary file 1 [file ijms-20-00738-s001.zip › Suppl files_rev/Supp Data_Text_Figs.pdf]

Supplementary Data for

Title: Deregulation of biologically significant genes and associated molecular pathways in the oral epithelium of electronic cigarette users

Authors: Stella Tommasi [1], Andrew W. Caliri [1], Amanda Caceres [1], Debra E. Moreno [1], Meng Li [2], Yibu Chen [2], Kimberly D. Siegmund [1], and Ahmad Besaratinia [1,*]

Affiliations: [1] Department of Preventive Medicine, USC Keck School of Medicine, University of Southern California, M/C 9603, Los Angeles, CA 90033, USA; [2] USC Libraries Bioinformatics Service, University of Southern California, NML 203, M/C 9130, Los Angeles, CA 90089

*Correspondence to: besarati@med.usc.edu

This PDF file includes:

Supplementary Text: Materials and Methods

Tables S1-S3

Figures S1-S2

Data processing and analysis for RNA-seq

RNA-seq data were processed and analyzed using Partek Flow version 7 (Partek Inc., St. Louis, MO). Briefly, raw sequencing reads were first trimmed from both ends (Phred QC score ≥ 20, minimum read length = 25 nt). Trimmed reads were then mapped to the human genome hg38 build using Star version 2.5.3a with default parameter settings [1]. Gencode 27 annotation [2] was used to quantify the aligned reads to genes/transcripts using Partek E/M method. Read counts per gene or transcript in all samples were normalized using Upper Quartile normalization [3], and analyzed for differential expression using Partek Gene Specific Analysis method. To ensure quality control, we set up the following inclusion/exclusion criteria: samples were included in the analysis if they had high sequencing depth (more than 15 million reads/sample) and high % reads aligned (greater than 50% reads aligned). Using these criteria, 83 of the 93 samples were included in the analysis; the excluded samples were 7 from the e-cig users and 3 from nonsmokers. Furthermore, we excluded any genes/transcripts with less than 10 reads in any sample among a data set. The differentially expressed gene (DEG) or transcript (DET) lists were generated for each comparison using the cutoff of $P < 0.005$ and fold changes greater than 1.5 in either direction, which corresponds to a false-discovery rate (FDR) -adjusted P-value of 0.1 in e-cig users and 0.07 in smokers. Subsequent functional analysis of the DEG lists was carried out using the Database for Annotation, Visualization and Integrated Discovery (DAVID) Bioinformatics Tool v.6.8 [4] and the Ingenuity Pathway Analysis (IPA®) (QIAGEN Bioinformatics, Redwood City, CA).

Cotinine measurement

Plasma cotinine was measured by a solid phase competitive enzyme-linked immunosorbent assay (ELISA) kit (Abnova Corp., Walnut, CA). Briefly, aliquots of standard controls and plasma

samples from the study subjects were loaded in triplicate (10 μl each) onto a 96-microwell plate pre-coated with a polyclonal antibody raised against cotinine. After adding a cotinine horseradish peroxidase enzyme (100 μl per well), the microplate was incubated for 1 hour at room temperature in the dark. Unbound cotinine and cotinine enzyme conjugate were washed off by rinsing the wells six times with distilled water (300 μl each wash). A chromogenic substrate (3,3',5,5'-Tetramethylbenzidine) was added (100 μl per well), and the plate was incubated for 30 minutes at room temperature. The reaction was terminated by adding a stop solution (100 μl per well), and absorbance was read at 450 nm using an iMark™ Mircroplate Absorbance Reader (BioRad Laboratories, Inc.). The assay sensitivity based on the minimum cotinine concentration required to produce a three-standard deviation from assay A0 is 1 pg/μl. The assay detection limit is 5 pg/μl. One-half of the detection limit (5 ÷ 2 = 2.5 ng/ml) was assigned to all samples with undetectable cotinine levels.

References

[1] A. Dobin, T.R. Gingeras, Mapping RNA-seq Reads with STAR, Current protocols in bioinformatics, 51 (2015) 11 14 11-19.

[2] J. Harrow, A. Frankish, J.M. Gonzalez, E. Tapanari, M. Diekhans, F. Kokocinski, B.L. Aken, D. Barrell, A. Zadissa, S. Searle, I. Barnes, A. Bignell, V. Boychenko, T. Hunt, M. Kay, G. Mukherjee, J. Rajan, G. Despacio-Reyes, G. Saunders, C. Steward, R. Harte, M. Lin, C. Howald, A. Tanzer, T. Derrien, J. Chrast, N. Walters, S. Balasubramanian, B. Pei, M. Tress, J.M. Rodriguez, I. Ezkurdia, J. van Baren, M. Brent, D. Haussler, M. Kellis, A. Valencia, A. Reymond, M. Gerstein, R. Guigo, T.J. Hubbard, GENCODE: the reference human genome annotation for The ENCODE Project, Genome research, 22 (2012) 1760-1774.

[3] J.H. Bullard, E. Purdom, K.D. Hansen, S. Dudoit, Evaluation of statistical methods for normalization and differential expression in mRNA-Seq experiments, BMC bioinformatics, 11 (2010) 94.
[4] W. Huang da, B.T. Sherman, R.A. Lempicki, Systematic and integrative analysis of large gene lists using DAVID bioinformatics resources, Nat Protoc, 4 (2009) 44-57.
[5] N. Cancer Genome Atlas, Comprehensive genomic characterization of head and neck squamous cell carcinomas, Nature, 517 (2015) 576-582.

Supplementary Figure legends

Figure S1. Comparison of the upstream regulators affected in both e-cig users and smokers relative to controls by IPA®. The heatmap visualizes the upstream regulators, including transcription factors and chemicals, whose activities were simultaneously affected in e-cig users and smokers.

Figure S2. Visualization of the network of deregulated genes converging on *TP53* gene in e-cig users and smokers relative to controls by IPA®. The tumor suppressor p53 gene (*TP53*) is the most frequently mutated gene in head and neck squamous cell carcinoma [5].

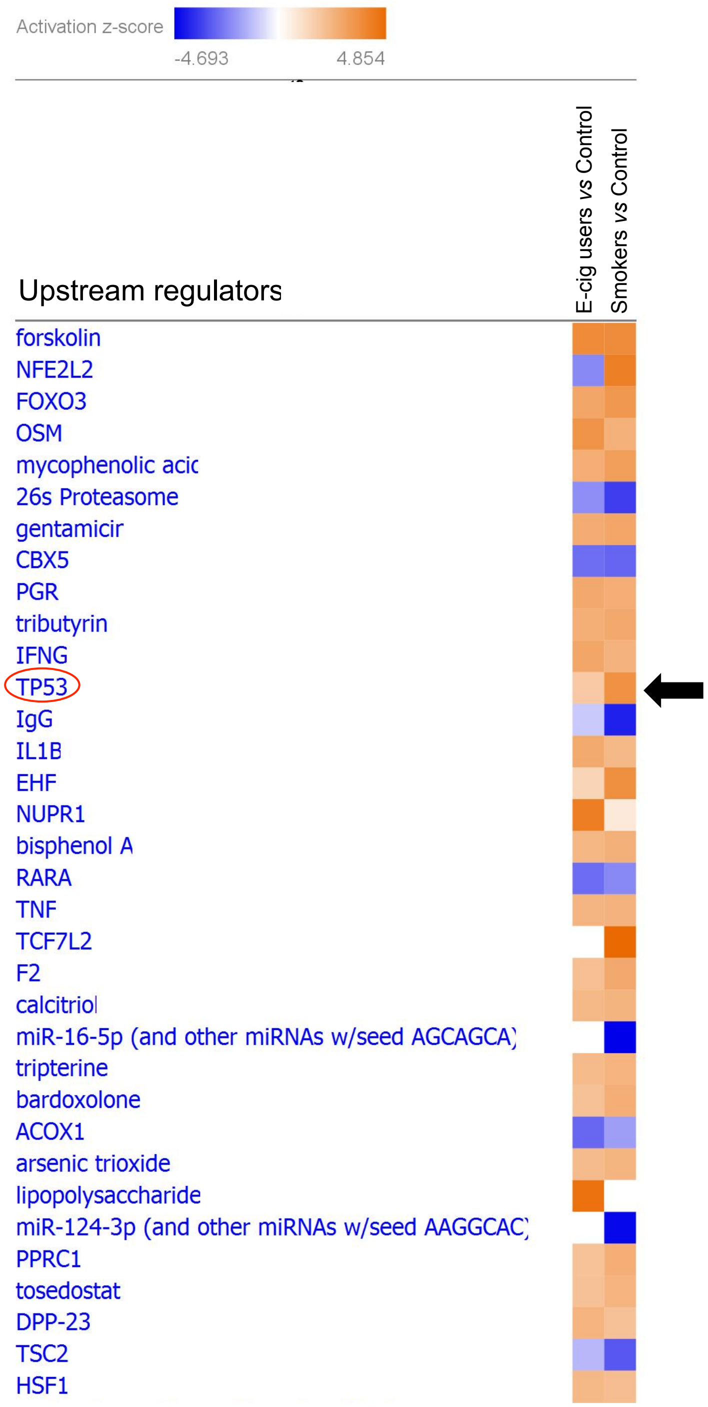
Activation z-score
-4.693
4.854
Upstream regulators
E-cig users vs Control
Smokers vs Control
forskolin
NFE2L2
FOXO3
OSM
mycophenolic acic
26s Proteasome
gentamicir
CBX5
PGR
tributyrin
IFNG
TP53
IgG
IL1B
EHF
NUPR1
bisphenol A
RARA
TNF
TCF7L2
F2
calcitriol
miR-16-5p (and other miRNAs w/seed AGCAGCA)
tripterine
bardoxolone
ACOX1
arsenic trioxide
lipopolysaccharide
miR-124-3p (and other miRNAs w/seed AAGGCAC)
PPRC1
tosedostat
DPP-23
TSC2
HSF1

Fig. S1

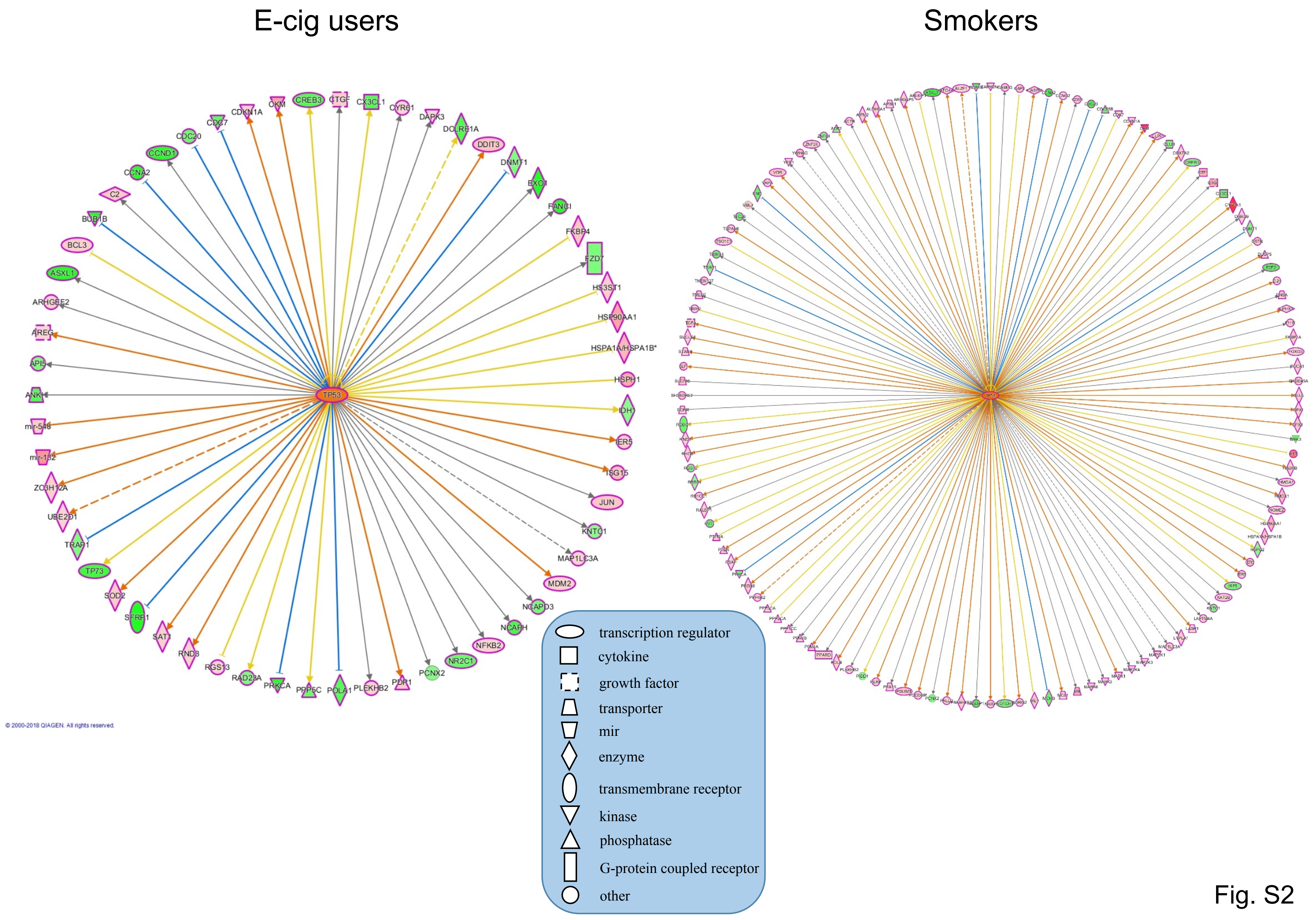
E-cig users
Smokers
TP53
CREB3
CTGF
CX3CL1
CYR61
DAPK3
DCLRE1A
DDIT3
DNMT1
EXO1
FANCI
FKBP4
FZD7
HS3ST1
HSP90AA1
HSPA1A/HSPA1B*
HSPH1
IDH1
IER5
ISG15
JUN
KNTC1
MAP1LC3A
MDM2
NCAPD3
NCAPH
NFKB2
NR2C1
PCNX2
PDP1
PLEKHB2
POLA1
PPP5C
PRKCA
RAD23A
RGS13
RND3
SAT1
SFRP1
SOD2
TP73
TRAP1
UBE2D1
ZC3H12A
mir-132
mir-548
ANKH
API5
AREG
ARHGEF2
ASXL1
BCL3
BUB1B
C2
CCNA2
CCND1
CDC20
CDC7
CDKN1A
CKM
© 2000-2018 QIAGEN. All rights reserved.
transcription regulator
cytokine
growth factor
transporter
mir
enzyme
transmembrane receptor
kinase
phosphatase
G-protein coupled receptor
other

Fig. S2
